# CASE REPORT Postoperative Use of the NormaTec Pneumatic Compression Device in Vascular Anomalies

**Published:** 2012-01-24

**Authors:** Simon G. Talbot, David Kerstein, Laura F. Jacobs, Joseph Upton

**Affiliations:** ^a^Department of Plastic Surgery, Children's Hospital, Boston, MA; ^b^NormaTec, Newton Center, MA

## Abstract

Arteriovenous malformations have a wide range of clinical presentations and an unfortunately unpredictable response to both nonsurgical and surgical intervention. The authors report on the surgical treatment of a 19-year-old man with a complex lower extremity arteriovenous malformation, previously unsuccessfully treated with numerous local sclerotherapy and interventional radiology embolization procedures leading to massive tissue necrosis and deep infection. The patient was definitively treated with wide excision of the necrotic tissue, coils, and arteriovenous malformation, but with preservation of the tibial nerve and vascular supply to the foot. Significant postoperative complications were prevented with the use of a novel dynamic compression device employing peristaltic pulse pneumatic compression.

Peripheral surgical wounds can be complicated by several common sequelae including operative site edema, distal limb edema, wound dehiscence, hemorrhage, hematoma, infection, tissue necrosis, and deep vein thrombosis.^1^ Modalities such as compression wraps, ice, and limb elevation have long been used in the postoperative period to reduce the incidence of these complications. In this case report, we describe the use of pneumatic compression to help improve postsurgical clinical outcomes. This particular case was especially challenging as it involved the surgical treatment of a complex lower extremity arteriovenous malformation that had failed numerous prior interventions spanning several years. Both the definitive surgical approach and the postoperative application of a unique pneumatic compression device that employs biomimicry to emulate physiologic forces by way of a peristaltic pulse waveform are discussed.

## CLINICAL REPORT

**Figure 1:**
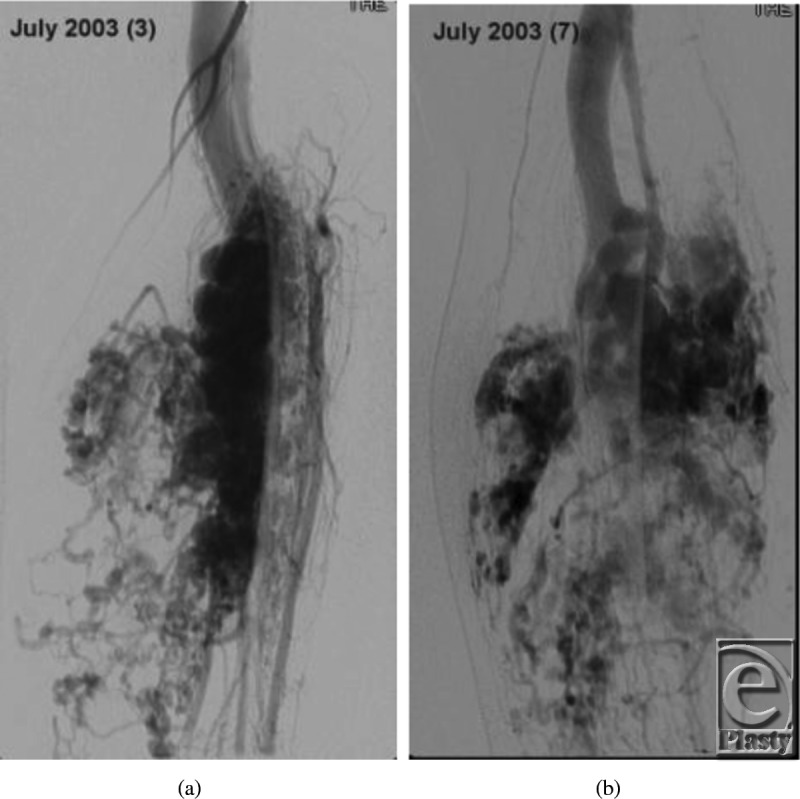
Preoperative angiogram demonstrating malformation with multiple coils.

**Figure 2:**
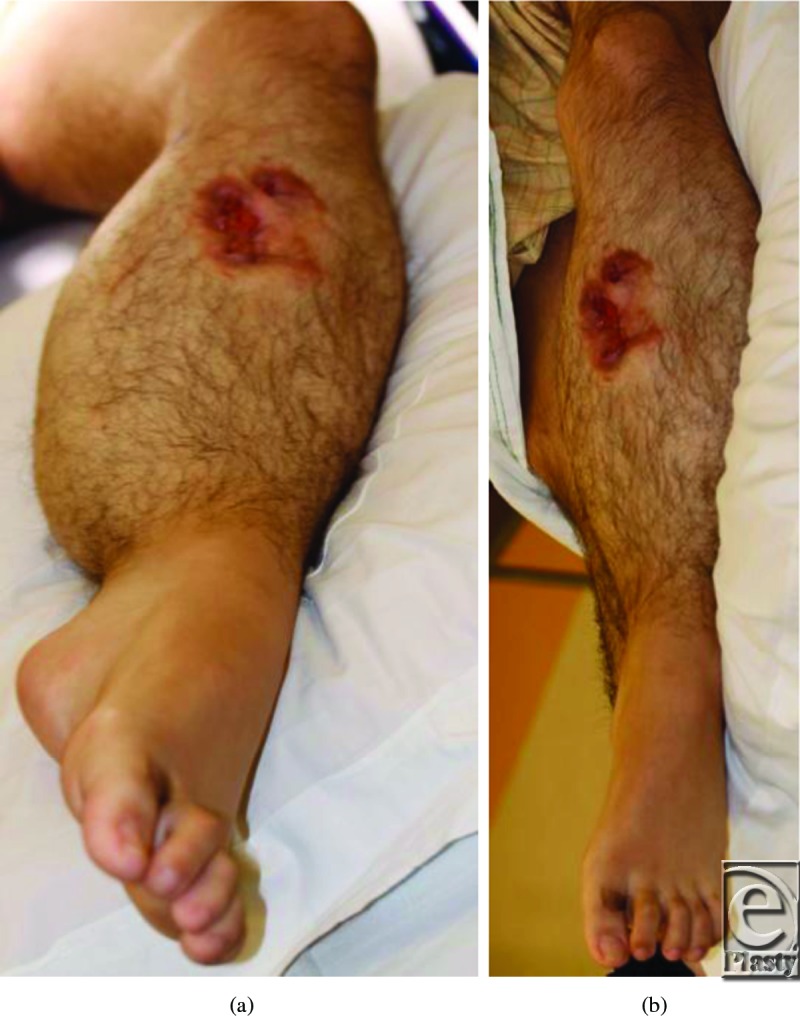
Preoperative wound with exposed coils.

Our patient is a 19-year-old man diagnosed with an arteriovenous malformation of the left lower leg before the age of 10 years from outside of the United States (Figs 1a and 1b). This progressed throughout his childhood and he had become essentially nonambulatory. He was initially treated with local sclerotherapy and embolization in England, starting at the age of 11 years and continuing with 8 additional embolization procedures over the course of 2 years. The embolization procedures were ineffective, and he was subsequently treated with 27 separate interventional radiological coiling procedures in the United States, the latest being performed in early 2010. The patient unfortunately then developed significant tissue necrosis with numerous exposed coil and subsequent deep infection. Skin grafting was attempted but failed, leaving him with a chronic abscess, significant limb edema, pain, foot drop, and severe restriction in his ambulation and activities of daily living (Fig 2). Multiple centers had recommended amputation of the limb.

**Figure 3:**
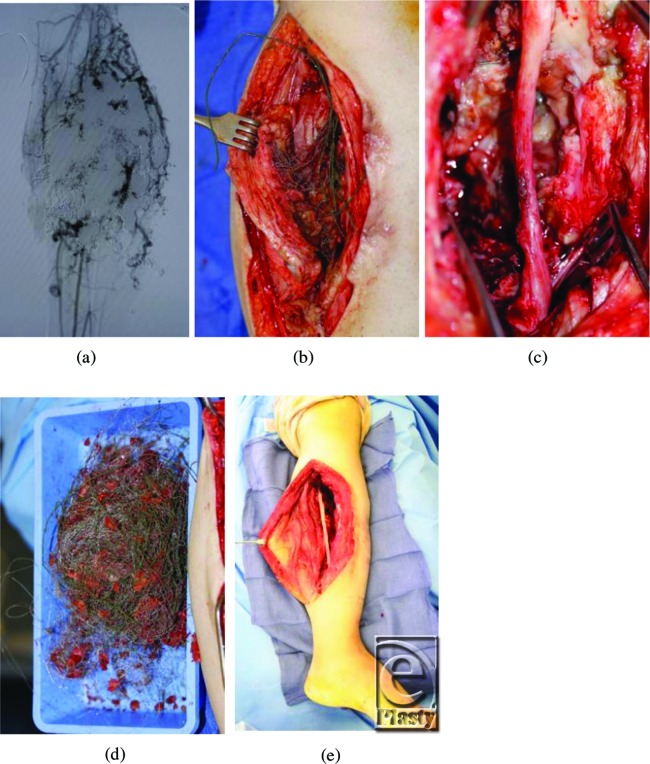
Coils were removed at surgery and the posterior tibial nerve was protected.

**Figure 4:**
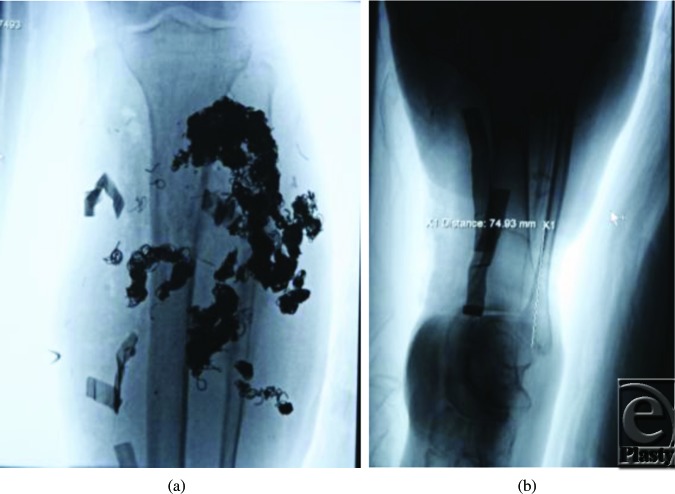
Angiogram after our first case demonstrating residual coils and vascular supply to the foot. A perforator joining the anterior and posterior circulations is marked 74 mm above the lateral malleolus.

In December 2010, he was referred to the Children's Hospital Boston Vascular Anomalies Clinic for evaluation. Three days after presentation to our clinic, he was taken to the operating room for wide debridement of the exposed coils, excision of the malformation including much of the deep posterior compartment of the lower leg, and direct closure of the wound. Under tourniquet control, the wound and sinus tracts were excised and large numbers of coils were removed. The posterior tibial nerve was exposed, preserved, and protected with a collagen nerve conduit (Fig 3). Given the extent of presenting infection, the wound was packed with Gelfoam and Surgicel, and a static compression dressing applied. A formal angiogram, obtained at this time to ascertain the vascular supply to the foot, showed trifurcation of the infrapopliteal vessels with embolized posterior tibial and peroneal arteries (Fig 4).

Three days later, the wound was further debrided including excision of the embolized soleus muscle and portions of the medial and lateral gastrocnemius muscle, with preservation of the perforator joining the anterior and posterior arterial systems. Achilles tendon lengthening was also performed to return the foot to a neutral position. Once again, Gelfoam was used to pack the wound and the leg was dressed with a static compression wrap. A planned delayed primary closure was performed with placement of two # 19 Blake drains 5 days after the initial procedure.

**Figure 5:**
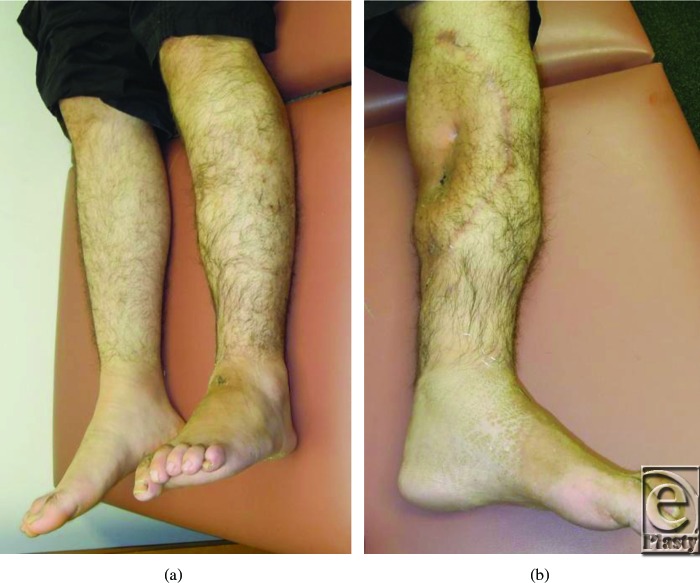
Postoperative appearance, with full wound healing and minimal residual edema.

Despite initial immobilization of the limb in an Air-Cast boot and elevation, the patient developed considerable lower leg edema with worsening pain. Given our positive experiences with peristaltic pulse pneumatic compression in patients with lymphedema, we elected to begin therapy with the NormaTec PCD (Pneumatic Compression Device) for up to 5 hours per day. Within 1 week, the left lower leg was significantly smaller in circumference with only a trace of edema present. The wound was under no tension, and drain output was minimal. The drains were removed and the patient began physical therapy to improve ambulation. He returned home with ongoing use of the PCD during his recovery. At 4 months, the patient was ambulating fully with decreasing opiate requirements, and all wounds were well healed (Fig 5).

## DISCUSSION

Although in use for more than 40 years in a broad array of clinical applications including deep vein thrombosis prophylaxis,^2^ healing of venous leg ulcers,^3^ treatment of lymphedema,^4^ and following orthopedic and vascular surgeries,^5^^-^8 the various methods of providing noninvasive pneumatic compression are not uniformly efficacious. For instance, modalities that solely provide compression to the plantar surface of the foot have been shown to be inferior to static compression in the prevention and treatment of edema following autologous femoropopliteal bypass surgery.^9^

The pneumatic compression device used in this case provides physiologic dynamic compression to the entire length of the treated limb. The NormaTec PCD comprises multicell sleeves that cyclically inflate and deflate with precisely calibrated pneumatic pressure in a patented peristaltic pulse waveform. The waveform employs a pulsing, gradient, and distal release compression pattern that mimics physiologic fluid dynamics. Pulsing compression simulates the skeletal “muscle pump,” pressure gradients between cells parallels the directionality of the distal to proximal flow created by 1-way venous and lymphatic valves, and the distal to proximal sequencing of compression coupled with distal release mimics the peristaltic compression wave found in lymphatic vessels.

The use of peristaltic pulse pneumatic compression has recently been reported in the treatment of secondary lymphedema after distal radius fracture with full functional outcome,^10^ and we are similarly using it for this purpose with excellent results. Pneumatic compression is thought to reduce edema^11^ by moving excess tissue fluid to the intravascular space and promoting venous and lymphatic return to the heart, leading to decreased tissue pressures and improved microcirculation,^12^ as well as closer tissue approximation within the surgical site.

While a single case cannot provide conclusions regarding new therapy, we believe that the NormaTec PCD was contributory in the uncomplicated healing of this complex wound, particularly in the setting of a high-flow vascular anomaly and swelling refractory to static compression and elevation. The impressive postsurgical outcome described earlier suggests the need for more rigorous investigation of peristaltic pulse pneumatic compression after surgery in comparison with standard modalities used to decrease postoperative edema and pain and to facilitate wound healing.
